# Using random forest machine learning on data from a large, representative cohort of the general population improves clinical spirometry references

**DOI:** 10.1111/crj.13662

**Published:** 2023-07-13

**Authors:** Kris Kristensen, Pernille H. Olesen, Anna K. Roerbaek, Louise Nielsen, Helle K. Hansen, Simon L. Cichosz, Morten H. Jensen, Ole Hejlesen

**Affiliations:** ^1^ Department of Health Science and Technology Aalborg University Aalborg Denmark; ^2^ Steno Diabetes Center North Denmark Aalborg Denmark

**Keywords:** clinical references, COPD, misdiagnosis, multiple linear regression, random forest, spirometry

## Abstract

**Introduction:**

Spirometry is associated with several diagnostic difficulties, and as a result, misdiagnosis of chronic obstructive pulmonary disease (COPD) occurs. This study aims to investigate how random forest (RF) can be used to improve the existing clinical FVC and FEV1 reference values in a large and representative cohort of the general population of the US without known lung disease.

**Materials and methods:**

FVC, FEV1, body measures, and demographic data from 23 433 people were extracted from NHANES. RF was used to develop different prediction models. The accuracy of RF was compared with the existing Danish clinical references, an improved multiple linear regression (MLR) model, and a model from the literature.

**Results:**

The correlation between actual and predicted FVC and FEV1 and the 95% confidence interval for RF were found to be FVC = 0.85 (0.85; 0.86) (*p* < 0.001), FEV1 = 0.92 (0.92; 0.93) (*p* < 0.001), and existing clinical references were FVC = 0.66 (0.64; 0.68) (*p* < 0.001) and FEV1 = 0.69 (0.67; 0.70) (*p* < 0.001). Slope and intercept for the RF models predicting FVC and FEV1 were FVC 1.06 and −238.04 (mL), FEV1: 0.86 and 455.36 (mL), and for the MLR models, slope and intercept were FVC: 0.99 and 38.56 39 (mL), and FEV1: 1.01 and −56.57‐57 (mL).

**Conclusions:**

The results point toward machine learning models such as RF have the potential to improve the prediction of estimated lung function for individual patients. These predictions are used as reference values and are an important part of assessing spirometry measurements in clinical practice. Further work is necessary in order to reduce the size of the intercepts obtained through these results.

## 1 INTRODUCTION

Chronic obstructive pulmonary disease (COPD) is one of the world's greatest health problems and is estimated to be the third foremost reason for death by 2020.^1^ The incidence of COPD in individuals over the age of 40 years is approximately 10%^2^; however, the precise incidence of COPD is difficult to estimate.^3^ In primary care, misdiagnosis of COPD occurs^4^; misdiagnosis covers underdiagnosis and overdiagnosis of COPD.^5^ Worldwide underdiagnosis ranged from 10–95%, whereas overdiagnosis ranged from 5% to 60%.^5^ A spirometry assessment should be conducted by trained and qualified personnel in a setting with a regular quality assurance program; misdiagnosis could be caused by inadequate quality assurance of spirometry.^6^ Spirometry is the most widely used lung function test in America and Europe, where forced expiratory volume in the first second (FEV1) and forced vital capacity (FVC) are essential in diagnosing and managing patients with COPD.^7^ In the case of the FEV1/FVC ratio being lower than the threshold value of 0.70, it will indicate the presence of COPD.^1^ However, spirometry is associated with several diagnostic difficulties. One of the difficulties is misdiagnosis caused by the wrong interpretation of the spirometry measurements made by healthcare professionals in primary care.^8^ In these cases, primary care tends to overdiagnose COPD,^9^ where general practitioners suggest an incorrect COPD diagnosis in approximately one‐third of the cases.^8^

The above‐mentioned errors, which can lead to misdiagnosis of COPD, indicate that an improvement in the quality control of spirometry measurements could be useful. Today, Danish healthcare professionals are assisted by a predicted lung function based on multiple linear regression (MLR) with age and height as predictors.^10^ However, these current reference estimates are not always precise and could lead to errors in diagnosing patients correctly.^11^ To minimize potential errors, an improvement of the existing prediction model would be beneficial. Furthermore, a prediction model could potentially be used in the development of a decision support system with the purpose of reducing the number of misdiagnoses in primary care.

Several studies developed equations for spirometry parameters. Among these studies are Mengesha et al.^12^ and Baltopoulos et al.^13^ Common to these studies are the multiple linear regression approach, the same predictors, similar results, no large representative cohort, and a large anthropometric diversity. There is a need for investigating different approaches in a large representative sample of both multiple ethnic groups and participants with large anthropometric diversity, which contributes to trying a different approach with additional predictors. A few studies tested other machine learning approaches, especially neural networks.^14^ A study by Boltis and Halkiotis^15^ managed to elevate the accuracy of the spirometry references from Baltopoulos et al.^13^ by using a neural network approach. The higher accuracy makes machine learning interesting to test on a representative cohort with large anthropometric diversity. Random forest (RF) has been chosen as the machine learning approach in this study due to more transparency compared with the neural network.

The aim of this study was to investigate the potential for RF to improve the existing clinical FVC and FEV1 reference values in a large and representative cohort of the general population.

## 2 MATERIALS AND METHODS

Data used in this study were extracted from the National Health and Nutrition Examination Survey (NHANES), which is freely available data collected from the US population. NHANES contained a large amount of different data types, including FVC, FEV1, demographics, dietary, examination, and laboratory data. The FVC and FEV1 measurements met the requirements of the American Thoracic Society and were acceptable measurements.^16^ The standards can be found in Miller et al.^7^

### 2.1 Participants

To select relevant data from NHANES, inclusion and exclusion criteria were established. Participants were excluded if they had unregistered predictors and if their FEV1/FVC ratio was below 0.7 or the lower limit of normal. Participants potentially suffering from lung disease were excluded due to the improvement of the existing clinical spirometry references addressed to healthy individuals.

Inclusion criteria

1. Participants with registered FVC and FEV1

Exclusion criteria

1. Participants with a FEV1/FVC ratio below 0.7 or the lower limit of normal
2. Participants with unregistered predictors

**FIGURE 1:**
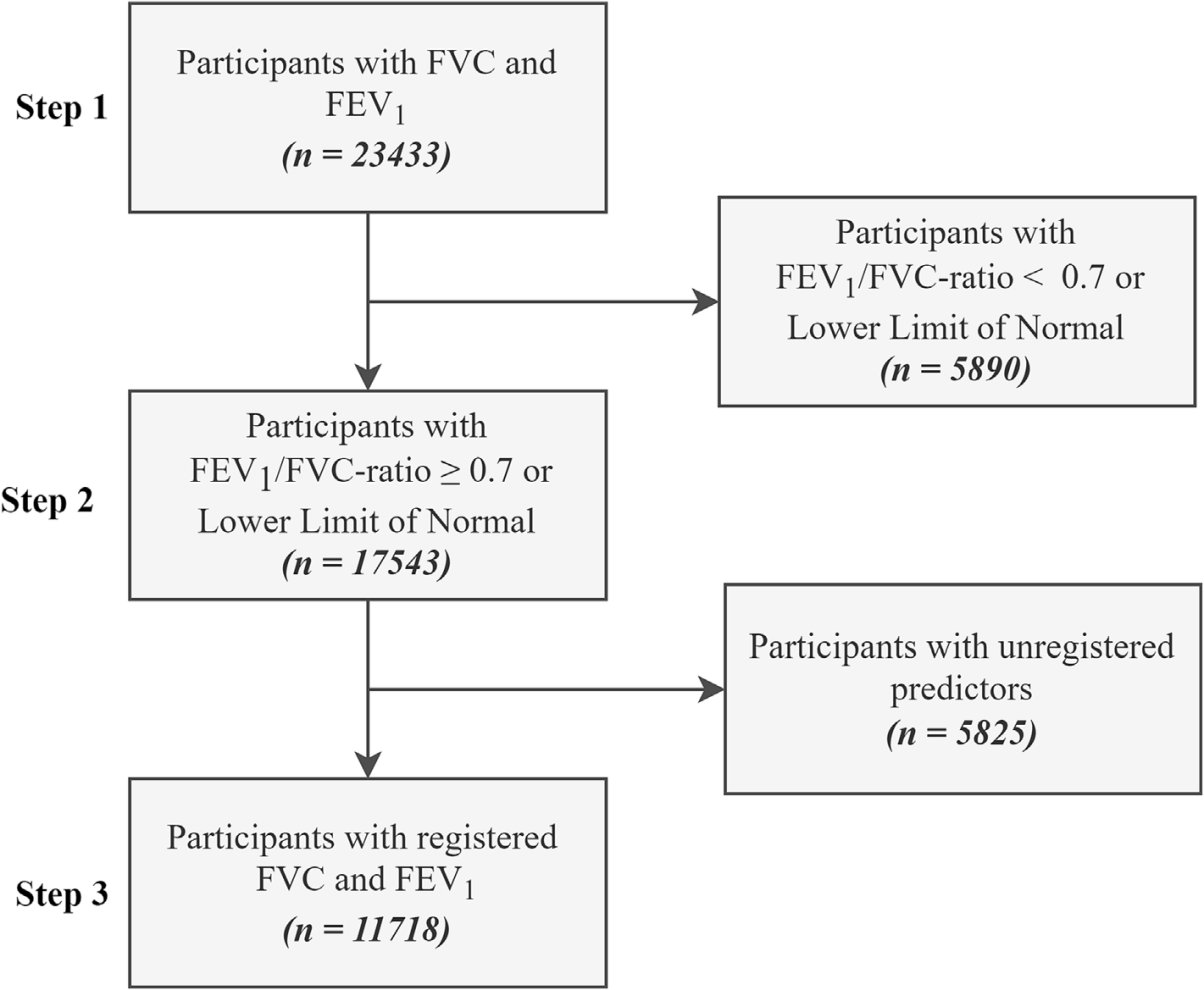
The selection of participants is shown in a flow diagram. Step 1 corresponds to the inclusion criterion, where the participants must have registered forced vital capacity (FVC) and forced expiratory volume in the first second (FEV1). Step 2 corresponds to the exclusion criteria that eliminate participants with an FEV1/FVC ratio below 0.7 or below the lower limit of normal. Step 3 corresponds to the exclusion criteria that remove participants with unregistered predictors.

The selection of relevant participants is illustrated in Figure 1.

### 2.2 Predictor selection

The selection of predictors was collected from studies^16^, ^17^, ^18^, ^19^ that illustrate various causes of misdiagnosis; these causes constituted the predictors of the final solution. The selected predictors were:

1. Gender
2. Ethnicity
3. Body mass index (BMI)
4. Smoking
5. Height
6. Weight
7. Waist measure
8. Diabetes
9. Systolic blood pressure
10. Diastolic blood pressure

The predictors would be tested to see whether a relation exists between them and FVC and FEV1, where the predictors that were not related to FVC and FEV1 would be excluded from further work on the development of a prediction model.

### 2.3 Model setup

**FIGURE 2:**
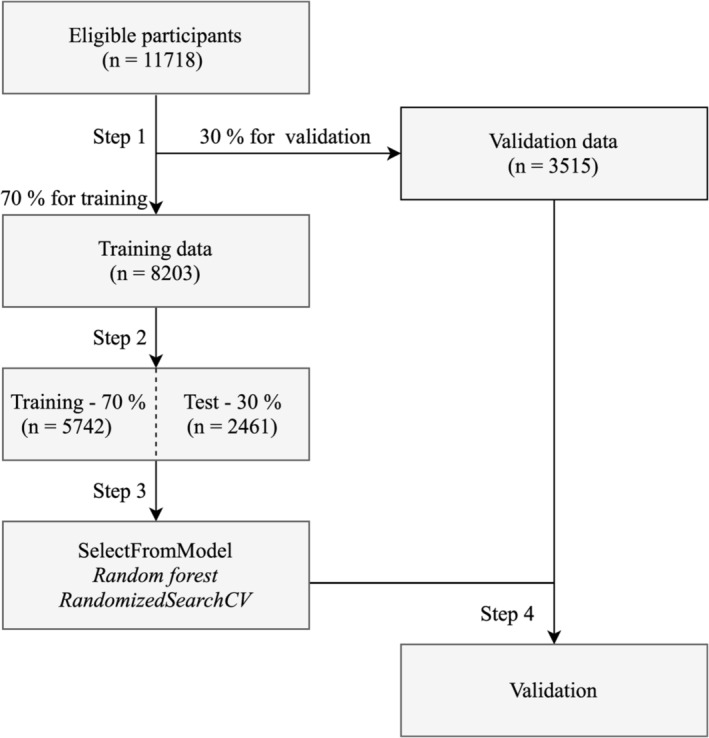
Stepwise development of a random forest (RF). Step 1: Participants were divided into 70% training data and 30% validation data. Step 2: Division of the 70% training data into an additional 70% and 30%. Step 3: The SelectFromModel was used to include predictors for the RF, and the RandomizedSearchCV function found the optimal value for each hyperparameter. Afterward, the RF model was created from the training data in step 2 and then tested on the 30% test data. This was repeated multiple times to minimize the probability of overfitting the model. Step 4: A final validation of the model was performed separately on the 30% validation data from step 1.

The study cohort was randomly divided into a training dataset, in which the RF was derived, and a validation dataset, in which the model was applied and tested to obtain an unbiased estimate of the model's performances. The training dataset was further divided into 70% training and 30% testing. The stepwise development of RF is shown in Figure 2. This procedure minimizes the potential for model overfitting.

#### 2.3.1 RF

RF was used as an improved prediction method for FVC and FEV1. The training, testing, and validation of the RF algorithm were performed with the free software machine learning library scikit‐learn. RF struggles to deal with categorical data such as ethnicity, which was categorized from one to five. RF placed more weight on ethnicity number five as it had a higher numerical value. To accommodate this, ethnicity was changed into five logic columns. Additionally, bootstrap aggregation is used as a method to calculate an average prediction across the decision trees.

RF is used as a supervised learning method where the prediction of FVC and FEV1 is made by mapping predictors. To select these predictors, a Python function was used to calculate the importance of each predictor and an average of them all, where predictors equal to or greater than the importance average were included in the RF model.

#### 2.3.2 Comparison to multiple linear regression

As reference to the RF model, MLR models were developed. MLR was chosen in this study because the existing Danish clinical spirometry references by Løkke et al.^20^ are made by this method. Three different MLR models were used in this study to assure an optimal reference for RF. The three models are the Danish clinical reference model, Mengesha et al.^12^ due to high prediction accuracy, and an improved MLR model with additional predictors developed in the current study.

#### 2.3.3 Validation of models

The RF and MLR models were lastly tested on the 30% validation data; see step 3 in Figure 2. The predicted values from each model were compared with the actual measured values from NHANES by plotting these in a comparison plot with the predicted values depicted on the x‐axis and the measured values on the y‐axis. Based on the R‐squared value from these plots, the accuracy of each model was evaluated.

## 3 RESULTS

Table 1 gives an overview of the participants' characteristics in the training and validation groups, where it is shown that the characteristics do not differ significantly between the two groups.

**TABLE 1:** A statistical overview of the demographic, questionnaire, and examination data for training and validation subjects. Numerical data were presented as a mean ± standard deviation and categorical data as a percentage of the total number of subjects. Further, the last column shows the *p*‐value for the Welch's t test made on the continuous variables and the chi‐squared test on the categorical variables.

	Training data	Validation data	*p*‐value
Number of subjects	8203	3515	
Average age (years)	38.8 ± 18.7	38.6 ± 18.7	0.137
Gender (%)			0.768
Male	50.5	49.4	
Female	49.5	50.6	
Ethnicity (%)			0.073
Mexican American	18.1	18.3	
Other Hispanic	11.4	12.2	
Non‐Hispanic White	39.0	38.0	
Non‐Hispanic Black	22.4	23.1	
Other ethnicity*	9.0	8.3	
Body mass index (kg/m^2^)	27.9 ± 6.8	28.2 ± 6.9	0.069
Smoking (yes %)	21.2	22.4	0.562
Height	167.4 ± 10.1	167.3 ± 10.2	0.481
Waist measure (cm)	94.7 ± 17.2	95.1 ± 17.4	0.585
Diabetes (yes%)	8.2	8.4	0.999
Blood pressure (mmHg)			
Systolic BP	119.0 ± 16.7	119.2 ± 16.1	0.523
Diastolic BP	68.4 ± 12.4	68.4 ± 11.8	0.869
FVC (mL)	3920 ± 1046	3930 ± 1039	0.869
FEV1 (mL)	3219 ± 870	3231 ± 867	0.329

Abbreviations: FEV1, forced expiratory volume in the first second; FVC, forced vital capacity.

The selected predictors for RF are shown in Table 2, which shows the total R‐squared values for each model in the training data, the 95% confidence interval, and values for the hyperparameters for FVC and FEV1.

**TABLE 2:** The table shows a test overview of the importance, total R‐squared values, and 95% confidence interval obtained for FVC and FEV1 for the selected predictors in the random forest model. Furthermore, the hyperparameters for each of the models are shown. The predictor non‐Hispanic Blacks were not selected for the FEV1 model.

Random forest—test data		
	FVC	FEV_1_
	Importance	Importance
Age	0.154	0.264
Non‐Hispanic Black	0.056	X
Height	0.546	0.464
Total R‐squared	0.85	0.86
CI	(0.84; 0.86)	(0.85; 0.87)
Number of trees	160	300
Min sample split	5	5
Min sample leaf	2	1
Max depth	32	25

Abbreviations: FEV1, forced expiratory volume in the first second; FVC, forced vital capacity.

**FIGURE 3:**
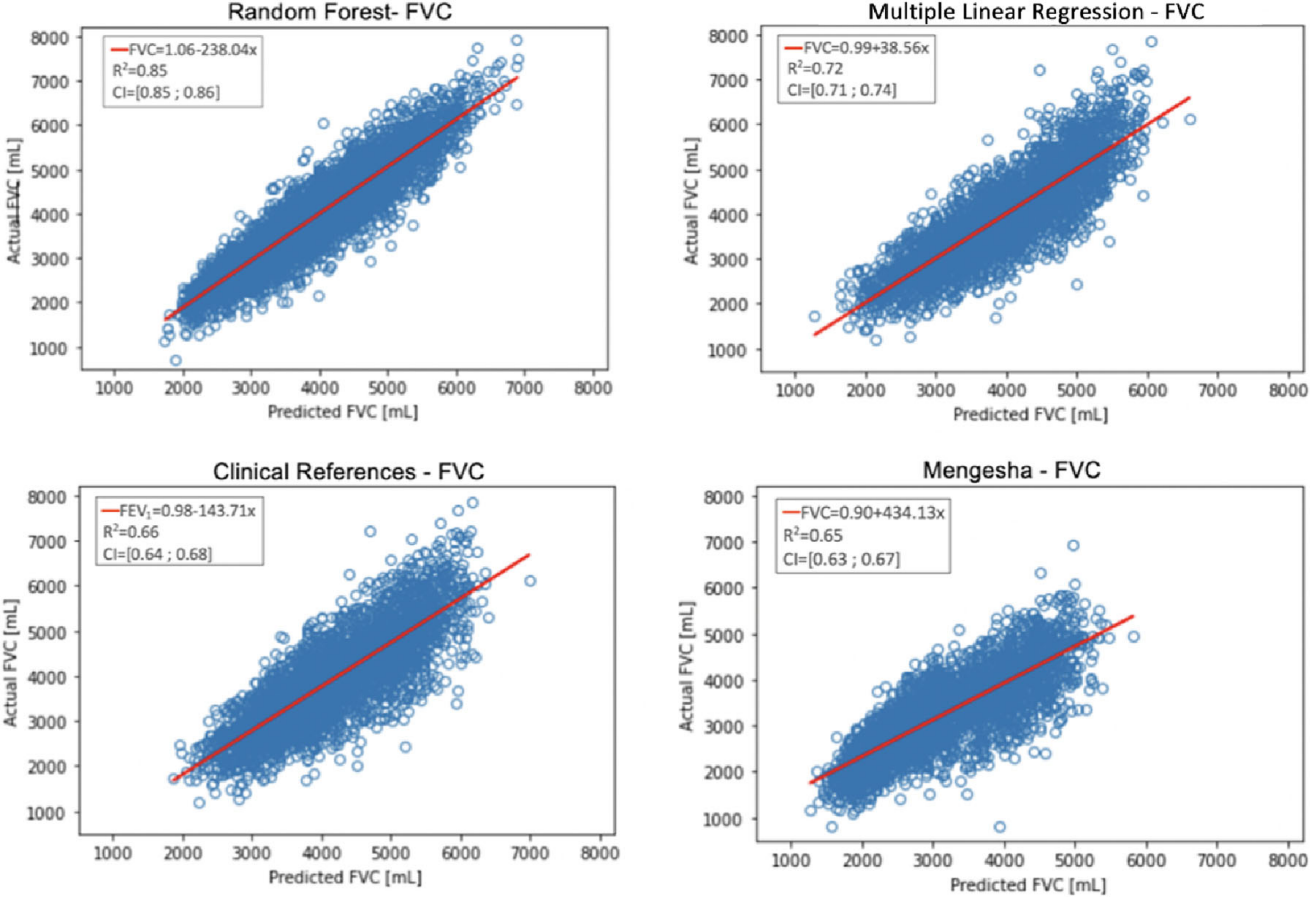
Illustrates the predicted forced vital capacity (FVC) compared with the actual FVC and the R‐squared with an additional 95% confidence interval. R‐squared for random forest (RF) equals 0.85 (0.84; 0.86), multiple linear regression (MLR) equals 0.72 (0.71; 0.74), Danish clinical references equals 0.66 (0.64; 0.68), and Mengesha et al. equals 0.65 (0.63; 0.67).

**FIGURE 4:**
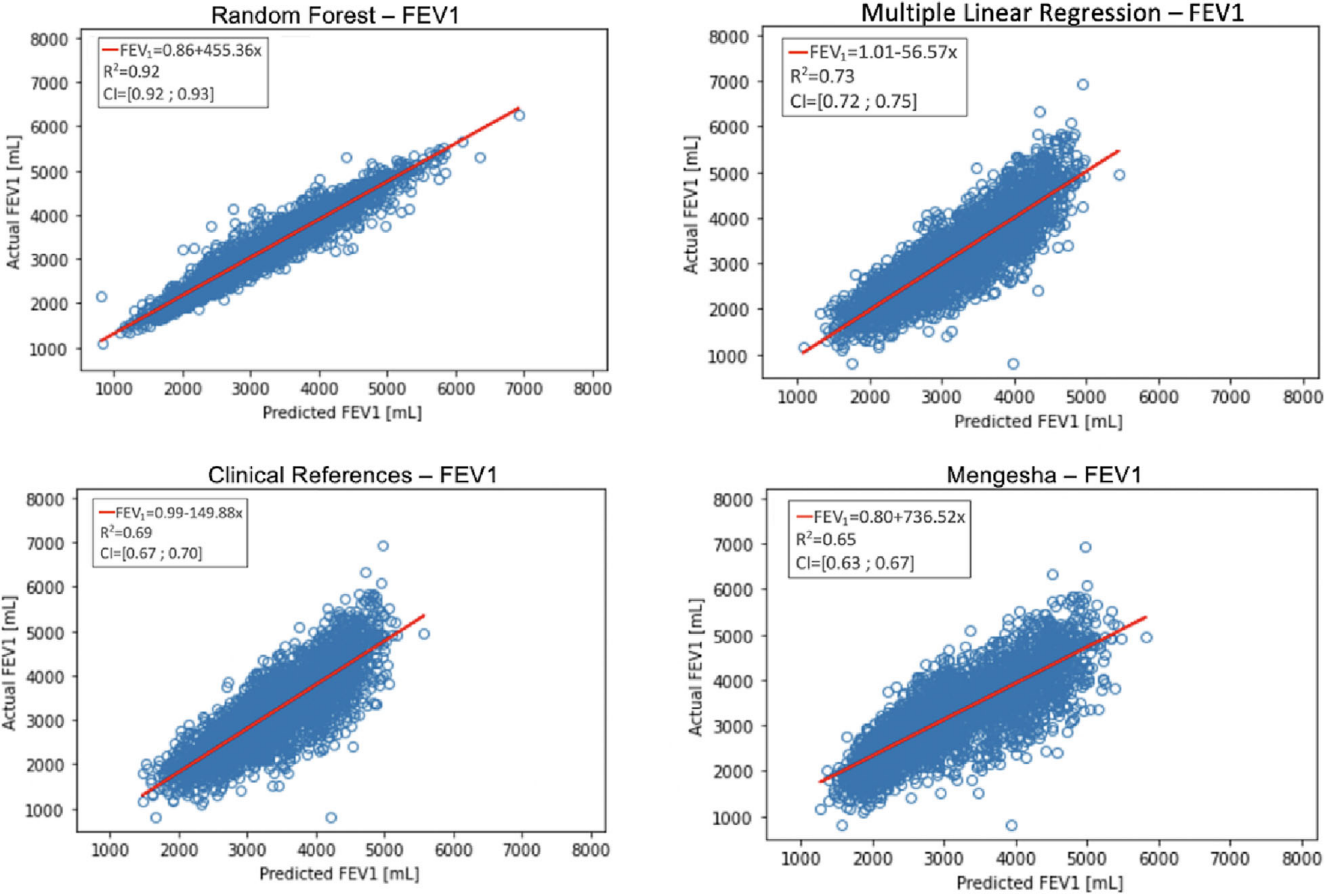
Illustrates the predicted forced expiratory volume in the first second (FEV1) compared with the actual FEV1 and the R‐squared with an additional 95% confidence interval. R‐squared for random forest (RF) equals 0.92 (0.92; 0.93), multiple linear regression (MLR) equals 0.73 (0.72; 0.75), Danish clinical references equals 0.69 (0.67; 0.70), and Mengesha et al. equals 0.65 (0.63; 0.67).

Validation of FVC and FEV1 for RF compared with the three types of MLR models used as references resulted in the comparison plots shown in Figures 3 and 4, where the estimations from each model were compared with the actual value from NHANES. Table 3 shows the slopes and intercepts for the comparison plots. For the models predicting FVC and FEV1, the slope and intercept were FVC: 1.06 and −238 (mL), FEV1: 0.86 and 455 (mL), and for the MLR models, the slope and intercept were FVC: 0.99 and 39 (mL), FEV1: 1.01 and −57 (mL). For the Danish clinical reference model, the slope and intercept were FVC: 0.98 and −144 (mL) and FEV1: 0.99 and −150 (mL). Likewise, for the Mengesha model, the slope and intercept were FVC: 0.90 and 434 (mL) and FEV1: 0.80 and 737 (mL).

**TABLE 3:** The table shows an overview of the slopes and intercepts for the comparison plots.

Model	Slopes	Intercepts (mL)
FVC		
Random forest	1.06	−238
Multiple linear regression	0.99	39
Clinical references	0.98	−144
Mengesha	0.90	434
FEV1		
Random forest	0.86	455
Multiple linear regression	1.01	−57
Clinical references	0.99	−150
Mengesha	0.80	737

Abbreviations: FEV1, forced expiratory volume in the first second; FVC, forced vital capacity.

**FIGURE 5:**
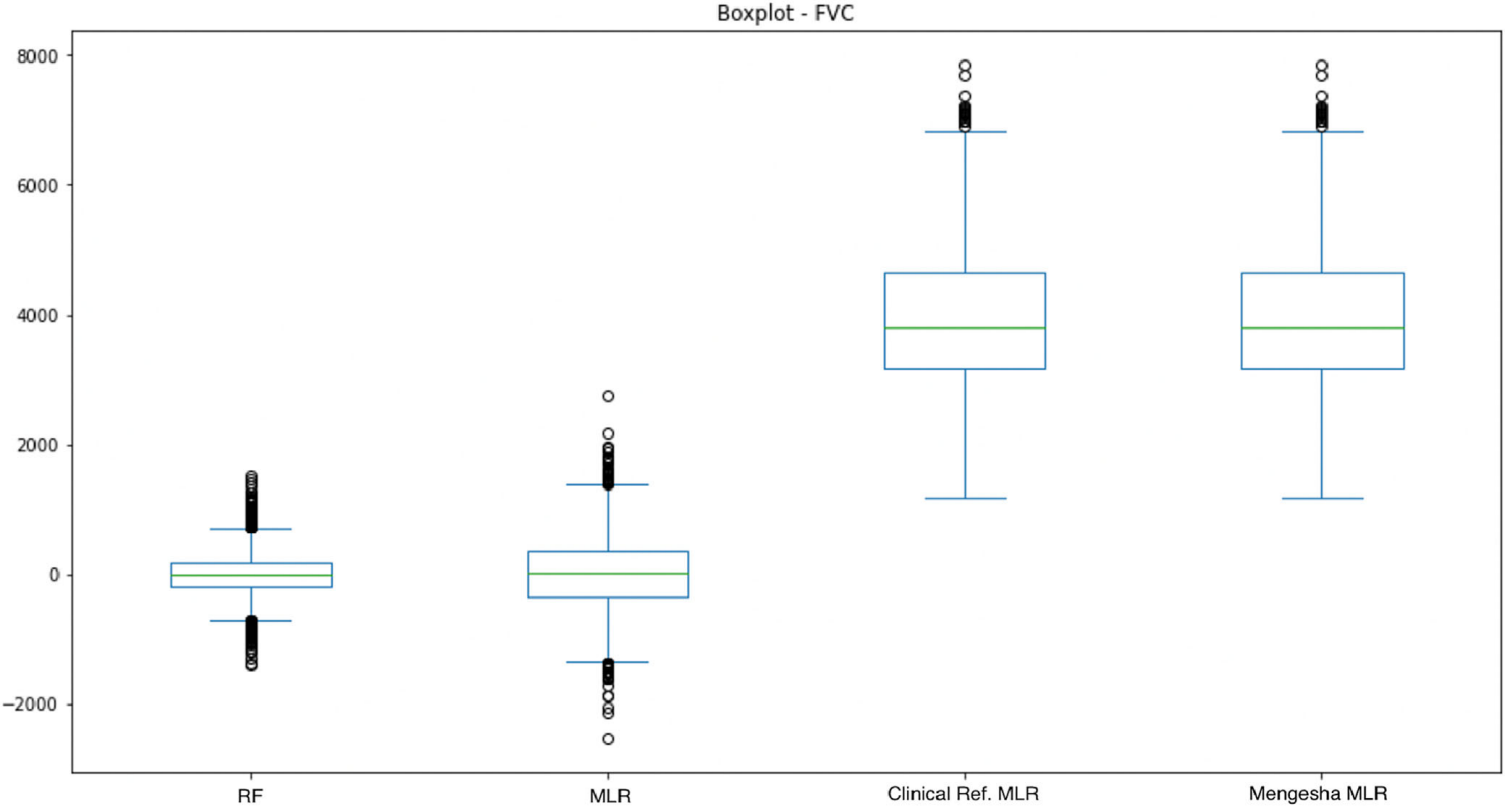
Illustrates a box plot of the four models for forced vital capacity (FVC).

**FIGURE 6:**
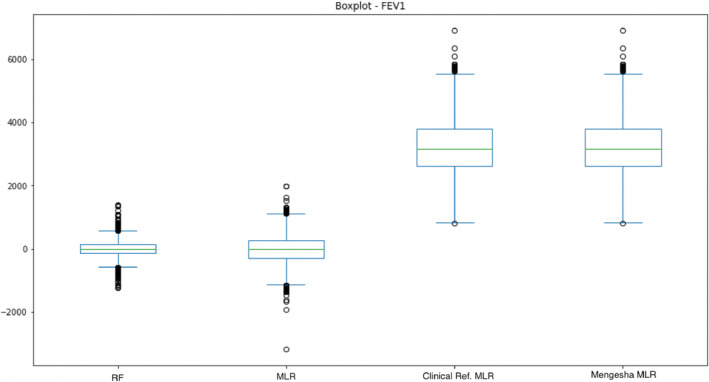
Illustrates a box plot of the four models for forced expiratory volume in the first second (FEV1).

Figures 5 and 6 show boxplots for the four models compared: the two models created in the study, Danish clinical references, and Mengesha et al.^12^ For both figures, it is seen that the median for both the developed RF and MLR models is lower than for the Danish clinical references and Mengesha et al.^12^ Further, the skewness in the data appears higher for both Danish clinical references and Mengesha et al.^12^

The results for FVC show an R‐squared value of 0.85 (0.85; 0.86) for RF, 0.72 (0.71; 0.74) for the developed MLR, 0.66 (0.64; 0.68) for Danish clinical references, and 0.65 (0.63; 0.67) for Mengesha et al.^12^

The results for FEV1 show an R‐squared value of 0.92 (0.92; 0.93) for RF, 0.73 (0.72; 0.75) for the developed MLR, 0.69 (0.67; 0.70) for Danish clinical references, and 0.65 (0.63; 0.67) for Mengesha et al.^12^

## 4 DISCUSSION

In our data, results from RF showed a higher R‐squared value for FVC and FEV1 than the MLR models: RF (0.85 and 0.92), MLR (0.72 and 0.73). Therefore, the machine learning method RF could be an improvement compared with MLR for clinical references. The slopes and intercepts for the FVC and FEV1 comparison plots, as shown in Table 3, differ from the slope with a max and minimum value of 0.80 to 1.06 and from the intercept −238 to 737 (mL). Based on the intercepts presented in Table 3, the MLR model for predicting FVC and FEV1 appears to be the most reasonable. However, the FVC and FEV1 models have considerably large intercepts, which can cause bias in the results, and future work should try to reduce the intercepts through the inclusion of more predictors.

To conclude on the performance of the RF models, a comparison to the study by Pramila et al.^21^ is completed. The R‐squared value for FEV1 is 0.96, which is higher than the value attained in the present study and is therefore a more accurate model for predicting FEV1. However, the study by Pramila et al.^21^ is limited because of its small group of subjects of 198 adults, which makes it difficult to transfer the results to other populations and countries. A strength of the presented study is the large and diverse population used to model the reference values. This includes a multi‐ethnic sample with a wide age span and both genders included.

The future purpose of the prediction models is to assist healthcare professionals in assessing whether the quality of spirometry is acceptable, which is why the models are based on healthy participants. A limitation in subjects like this may exclude certain types of a population, which results in poor prediction of this part of the population.

A limitation is that the data used in this study was limited to the US, so it is not known if these results are representative of other countries. As the population in the US is of a bright variation of ethnicity and the used cohort is large it is expected that the data can be used to test the Danish clinical reference models.

To further conclude on the improved MLR models, a comparison to the Danish clinical reference models was accomplished. The R‐squared values for the developed MLR model were FVC = 0.72 (0.71; 0.74) and FEV1 = 0.73 (0.72; 0.75), whereas the estimates from the Danish clinical reference models were FVC = 0.66 (0.64; 0.68) and FEV1 = 0.69 (0.67; 0.70). This comparison shows a more accurate estimation of FVC and FEV1 using the improved MLR model in the current study. The improved accuracy shows the potential of ML models and how they can be used to improve healthcare in general. The advantage of RF compared with other ML models is transparency. RF is a more see‐through model, which makes it easier for healthcare practitioners to understand both the model and the result and is therefore a better fit for healthcare. This improvement in accuracy could be explained by the inclusion of additional predictors in the improved MLR models, which included 10 predictors, whereas the Danish clinical reference models included four predictors. However, all of these additional predictors are easily obtainable and could be implemented in the clinical procedure of assessing spirometry.

However, whether the contribution from these additional predictors is enough to include is debatable. For example, it takes time for the healthcare professional to take blood pressure, and even then, the measure can be elevated due to white‐coat syndrome.^22^ The benefits of this predictor are limited due to its disadvantages, which is why blood pressure could have been excluded. Predictors such as blood pressure and waist measurements contribute little to the model and are time‐consuming due to measurements, which is why predictors like these are not nearly as relevant.

A limitation of the present study could be the exclusion of predictors. The selection method used for RF in the present study finds the importance of every predictor and then excludes the predictors that are under the average importance. This can be a limitation due to some of the excluded predictors still contributing to the model even if they are under the average. A solution to this can be found by investigating other threshold values for including more predictors, which may contribute to the models' accuracy.

An additional limitation of RF is the cross‐validation method, which randomly finds the best bid for the size of the hyperparameters. In the present study, the number of trees and depth are decreased to make RF faster without compromising accuracy. Changes in the other hyperparameters, such as minimum sample leaf and mean sample splits, can potentially contribute to the prediction models' speed as well, which should be further investigated.

Further work includes the implementation of the models as a tool with a user interface to support the healthcare professional in their decision of whether a performed spirometry is of good quality. By using the prediction model with the highest accuracy, it is possible to compare the current patient's FVC and FEV1 to the values that, according to the model, should correspond to a patient with the same values for the predictors. Knowing the theoretic values can give the physician further knowledge for the decision, which can cause the healthcare professional to make a more informed decision in order to decide whether or not a measurement is correct. If the actual measured values are nearly the same as predicted, it indicates that the measurement is of good quality and therefore should be approved. In contrast, if the measured value differs from the predicted value, it indicates that something in the measurement has gone wrong or that the patient suffers from a lung function disease. In this case, further measurements are needed to decide whether the measurement is correct.

## 5 CONCLUSION

Based on the results presented in this study, the developed RF model, as well as the developed MLR model, outperforms the current Danish clinical reference model regarding estimated lung function for individual patients. These findings indicate that a clinical model for predicting FVC and FEV1 can be advantageously based on RF. However, further work with these models is necessary to reduce the size of the intercepts.

## CONFLICT OF INTEREST STATEMENT

The authors declare no conflicts of interest.

## Data Availability

Research data are not shared.
